# Identification of robust and abundant reference transcripts for EV mRNA cargo normalization

**DOI:** 10.1016/j.vesic.2025.100065

**Published:** 2025-06

**Authors:** Antje M. Zickler, Radosław Grochowski, André Görgens, Erik Bäcklin, Maximilian Kordes, J.-Matthias Löhr, Joel Z. Nordin, Samir EL Andaloussi, Daniel W. Hagey

**Affiliations:** a Division of Biomolecular and Cellular Medicine, Karolinska Institutet, ANA Futura, Huddinge, 14152, Stockholm, Sweden; b Department of Cellular Therapy and Allogeneic Stem Cell Transplantation (CAST), Karolinska University Hospital, 141 86, Stockholm, Sweden; c Karolinska ATMP Center, Karolinska Institutet, ANA Futura, Huddinge, 14152, Stockholm, Sweden; d Center for Clinical Cancer Studies, Karolinska University Hospital, 17176, Stockholm, Sweden; e Department of Clinical Science, Intervention and Technology, Karolinska Institutet, Huddinge, 14152, Stockholm, Sweden; f Department of Clinical Immunology and Transfusion Medicine (KITM), Karolinska University Hospital, 14186, Stockholm, Sweden

**Keywords:** Extracellular vesicles, mRNA, Transcript, Standards, Normalization, RNA-Sequencing, qPCR

## Abstract

Extracellular vesicles (EVs) have been investigated intensively because of their potential as biomarkers of disease and their versatility as bioengineered therapeutic nanoparticles. EVs carry diverse biomolecular cargo, but absolute quantification has been challenging due to a lack of established molecular standards. Reliable identification of these has proven difficult owing to a scarcity of standardized global data sets spanning the heterogeneity of EV subtypes and cell sources. To identify reference messenger RNA (mRNA) transcripts, we analyze oligo-dT primed RNA-sequencing data from EVs originating from twelve different cell sources isolated using differential centrifugation followed by ultrafiltration. We identify 11 transcripts that are shared amongst the 50 most abundant in EVs from all of these cell sources. Following RT-PCR and deeper sequencing analysis, five transcripts warranted further investigation as molecular standards: TMSB4X, ACTB, GAPDH, VIM, and FTL. As such, we subjected the RT-qPCR results from two independent oligo-dT cDNA synthesis methods to stability assessment using the RefFinder analysis tool, conducted a proof-of-concept normalization on the levels of the variably expressed gene RAB13 and compared quantification of engineered mRNA loading with that of digital PCR. We confirmed the EV association of reference transcripts with EVs by performing gradient centrifugation followed by RT-qPCR and full-length mRNA analysis. To judge the applicability of these genes as reference transcripts for biomarker studies, we performed RNA-sequencing on EVs isolated from plasma by differential ultracentrifugation, and four other minimally processed biofluids. These findings confirm the applicability of these genes as molecular standards for EV-mRNA analysis and will aid in the standardization of EV research by establishing molecular reference genes that can be employed in diverse contexts.

## 1 Introduction

Extracellular vesicles (EVs) are lipid bilayer-enclosed natural nanoparticles released by all cells. EVs carry a diverse range of biomolecules, including proteins, lipids, and nucleic acids, and by transferring this cargo between cells they play an important role in cell-cell communication. Therefore, as key mediators of physiological and pathological processes EVs hold great potential as biomarkers, while their capacity to be bioengineered makes them promising natural therapeutic nanoparticles.^1^^,^^2^

Among EV cargoes, messenger RNA (mRNA) is of particular interest because each one can be translated into multiple functional proteins in recipient cells, potentially influencing cellular behavior.^3^ Consequently, accurate quantification of EV-derived mRNA is crucial for understanding its functional role or therapeutic impact, and for the identification of clinically relevant biomarkers.

Reverse transcription quantitative polymerase chain reaction (RT-qPCR) is the most widely used technique for the quantification of specific mRNA molecules. However, the reliability of RT-qPCR data depends on appropriate primer design and stable reference transcripts for normalization, commonly known as housekeeping genes.^4^ Suitable reference transcripts in EV research must exhibit consistent abundance across different experimental conditions, EV sources, and treatment groups to ensure accurate normalization and meaningful interpretation of mRNA levels between EV samples.^5^^,^^6^ To date, only a limited number of studies have addressed the topic of EV reference mRNA identification.^7^^,^^8^ Instead, current recommendations rely on the use of absolute quantification, indirectly by RT-qPCR standard curves or directly by digital PCR.^4^^,^^9^, ^10^, ^11^

The selection of appropriate reference genes for EV studies is particularly challenging due to the heterogeneous nature of EV populations derived from a single source, which can differ in their RNA content and composition.^12^ Although commonly used in cellular RNA studies,^5^ traditional housekeeping genes may not be suitable for EV-derived mRNA due to their variability or the biology of loading specific mRNAs into EVs. Therefore, identifying stable and universally applicable reference transcripts for EV research is essential to standardize RT-qPCR results in EV research.^4^

**Fig. 1:**
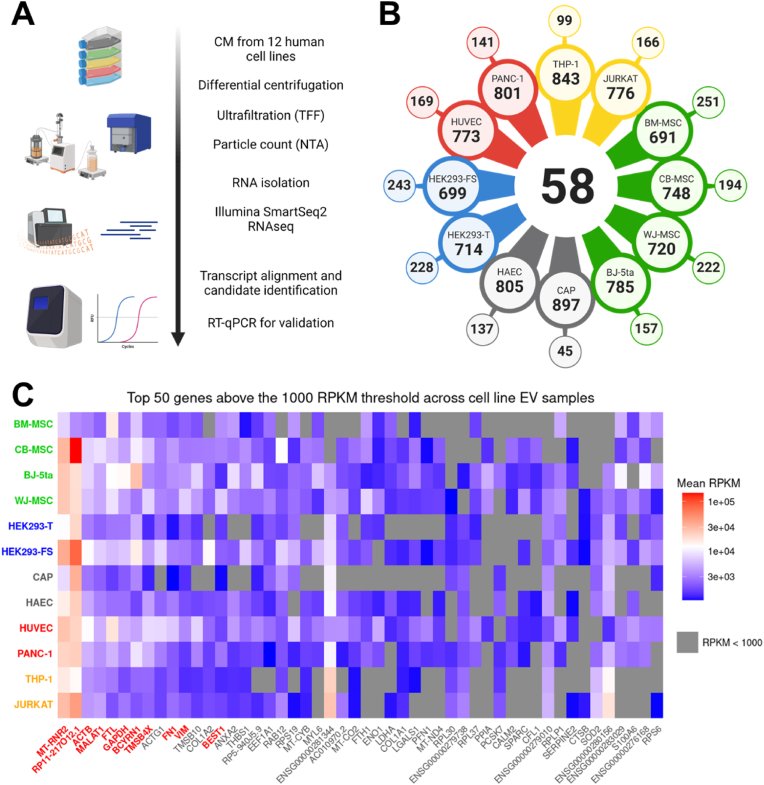
Identification of common mRNA transcripts associated with EVs from 12 cell sources. **(A)** Workflow of EV preparation, RNA sequencing (RNA-seq) and analysis, and validation of reference transcripts by RT-qPCR. Figure created using BioRender. **(B)** Top-ranked 1000 transcripts per EV sample were analyzed for commonly shared, partially shared, and unique genes. Central number: number of commonly shared transcripts across all EV samples. Large colored circles: number of transcripts of indicated EV sample partially shared with others. Small colored circles: number of unique transcripts of indicated EV sample. **(C)** Rank-sum ordered top 50 transcripts with average expression values of 1000 RPKM. Top 11 transcripts with common abundance and robust RPKM values > 1000 are marked in red. Grey tiles indicate EV samples with RPKM <1000 for the respective gene.

In this study, we aimed to address this challenge by analyzing RNA-sequencing (RNA-seq) data from EVs derived from multiple sources to identify highly and stably expressed reference genes (Fig. 1A). We then validated five candidate reference genes using RT-qPCR to assess their suitability for transcript normalization in EV-related studies. Finally, we verified their abundance in EVs from blood plasma, a complex biological fluid important in clinical settings. Our findings provide critical insights into the selection of reference genes for EV-derived mRNA quantification and contribute to the development of more reliable methodologies for studying the functional and diagnostic potential of EVs.

## 2 Material and methods

### 2.1 EV source cell culture

Cell lines were cultured in the following media: Immortalized, human bone marrow and umbilical cord derived mesenchymal stromal cells (hTERT + MSCs) were cultured in MEM-α Modification Medium (containing L-glutamine; Thermo Fisher Scientific) supplemented with 5 ng/ml of bFGF (Sigma, F0291). Wharton's jelly mesenchymal stem cells were cultured in DMEM-low glucose (containing Glutamax-I and sodium pyruvate; 1 g/L Glucose; Invitrogen), BJ-5ta fibroblast cells (Fibroblast immortalized with TERT) were cultured with 4:1 mixture of Dulbecco's medium (containing 4 mM L-glutamine, 4.5 g/L glucose and 1.5 g/L sodium bicarbonate) and Medium 199 (0.01 mg/ml Hygromycin B/10687010, Thermo Fisher Scientific), HUVEC were cultured in Vascular Cell Basal Medium (PCS-100-030, ATCC), supplemented with Endothelial Cell Growth Kit-VEGF (PCS-110-041, ATCC), PANC-1 cells were cultured in DMEM/F12 (containing 2.5 mM L-Glutamine, 15 mM HEPES), HEK293-T cells were cultured in DMEM (containing Glutamax-I and sodium pyruvate; 4.5 g/L Glucose; Invitrogen), HEK293 Freestyle suspension cells (HEK293-FS; ThermoFisher Scientific) were cultured in FreeStyle 293 Expression Medium (Thermo Fisher Scientific), and CAP amnionic fluid cells were cultured in serum-free Protein Expression Media (Thermo Fischer Scientific) with Glutamax-I and 2 μg/μl Puromycin, in 125 mL polycarbonate Erlenmeyer flasks (Corning) in a shaking incubator (Infors HT Minitron) according to the manufacturer’s instructions. HUAC amniotic epithelium, which were isolated from human placentae as previously described^13^ were cultured in Dulbecco's medium (containing 4 mM L-glutamine, 4.5 g/L glucose and 1.5 g/L sodium bicarbonate) supplemented with non-essential amino acid, beta-mercaptoethanol, and 5% Stemulate (Cook Regentec). THP-1 monocyte and Jurkat T-cell lines were cultured in RPMI-1640 medium (containing Glutamax-I and 25 mM HEPES, Invitrogen). Unless indicated otherwise, all cells were supplemented with 10% FBS (Invitrogen) and 1X Antibiotic-Antimycotic (Anti-Anti) (Thermo Fisher Scientific). All cell lines were grown at 37 °C, 5% CO_2_ in a humidified atmosphere and regularly tested for the presence of mycoplasma.

### 2.2 EV production and purification

For EV harvesting, cell culture-derived conditioned media (CM) was changed to OptiMEM (Invitrogen) 48 h before harvest, as described before.^14^ Unless indicated otherwise, all CM samples were directly subjected to a low-speed centrifugation step at 500×*g* for 5 min followed by a 2,000×*g* spin for 10 min to remove larger particles and cell debris. Pre-cleared cell culture supernatant was subsequently filtered through 0.22 μm bottle top vacuum filters (Corning, cellulose acetate, low protein binding) to remove any larger particles. EVs were prepared by tangential flow filtration (TFF). For the TFF EV preparation, pre-cleared CM was concentrated using the KR2i system (SpectrumLabs) equipped with modified polyethersulfone (mPES) hollow fiber filters with 300 kDa membrane pore size (MidiKros, 370 cm2 surface area, SpectrumLabs) at a flow rate of 100 ml/min (transmembrane pressure at 3.0 psi and shear rate at 3700 s^−1^) as described previously.^14^ Amicon Ultra-0.5 10 kDa MWCO spin-filters (Millipore) were used to concentrate the sample to a final volume of 100 μl. EV samples were stored at −80 °C in PBS-HAT buffer.^15^

### 2.3 EV production for differential mRNA loading assessment

HEK293-T cells stably expressing murine Ox40L (mOx40L) mRNA constructs were generated using the ϕC31 integrase system (System Biosciences) as published previously by our group.^16^ The cells express mOx40L mRNA from a genomically inserted expression cassette. As described previously,^16^ the mRNA sequence carries a 3′end target sequence compatible to binding by the engineered RNA-binding domain PUFe.^16^ To specifically load mOx40L mRNA into EVs, the cells were transfected with plasmid DNA expressing the RNA-binding domain PUFe fused to an EV sorting domain, CD63. As a non-loading control, cells were transfected with plasmid DNA encoding for CD63-NBC, with NBC as incompatible RNA-binding domain unable to bind to the PUFe target sequence.^16^

For loaded or control EV production, mOx40L mRNA-expressing cells were seeded in 150-mm cell culture dishes one day prior to reach a confluency of 70% at the time of transfection. Cells were transfected using PEI Max 40K (Polysciences) at a DNA:PEI ratio of 1:3 (w/w). Transfection complexes were allowed to form in serum-free defined medium (OptiMEM, Gibco), and added dropwise to the cells. After 4 h, transfection medium was removed and changed to OptiMEM as EV production medium as previously described.^17^

After 48 h, conditioned medium was collected and subjected to one of our streamlined differential centrifugation and ultrafiltration protocols for EV isolation. Briefly, medium was spun at 700×*g* for 5 min to remove residual cells, then at 2,000×*g* for 10 min to remove cell debris. The centrifuged medium was then filtered through a polyethersulfone (PES) membrane filter with 0.22 μm pore size (TPP). Next, one biological replicate of both conditioned media (loaded and control) was subjected to ultracentrifugation, while the other one was subjected to TFF/UF as described above. Ultracentrifugation was performed at 100,000×*g* for 90 min at 4 °C in a Beckman Coulter Optima XP Ultracentrifuge. Final EV preparations were kept in sterile PBS-HAT buffer for storage at −80 °C.^15^

### 2.4 Nanoparticle tracking analysis

Nanoparticle tracking analysis (NTA)^18^ was applied to determine particle size and concentration of all samples using the NanoSight NS500 instrument equipped with NTA 2.3 analytical software and an additional 488-nm laser. The samples were diluted in 0.2 μm filtered PBS to an appropriate concentration before being analyzed. At least five 30-s videos were recorded per sample in light scatter mode with a camera levels of 11–13. Software settings for analysis of scatter particles were kept constant for all measurements (screen gain, 10; detection threshold, 7). The analysis was performed with the screen gain at 10 and detection threshold at 7 for all measurements.

### 2.5 Multiplex bead-based EV flow cytometry assay

EV surface markers (CD9, CD63, CD81) were assessed on both cell culture-derived and plasma-derived EV samples by multiplex bead-based EV flow cytometry (MACSPlex EV Kit IO, Miltenyi Biotec) as described before.^19^ Briefly, prepared EV samples (assay input dose: 1x10^9^ NTA-based particles) were diluted with MACSPlex buffer to a final volume of 60 μl and incubated over-night with 10 μl MACSPlex Exosome Capture Beads on an orbital shaker at room temperature (RT) in the dark. Beads were washed with MACSPlex buffer, and then 4 μl of APC-conjugated CD9, CD63 and CD81 detection antibodies were added to each sample in a total volume of 135 μl. Following incubation for 1 h on an orbital shaker at RT, samples were washed again and incubated for another 15 min before a final washing step was performed. Incubation and washing steps were performed in 0.22 μm filter plates. Final samples were resuspended in 150 μl MACSPlex buffer and acquired on a MACSQuant Analyzer 16 flow cytometer (Miltenyi Biotec). Data was analyzed with FlowJo software version 10.9.0 and expressed as log10-transformed fold change values over respective non-EV containing buffer controls as described before.^20^ Heatmaps were generated with Morpheus (https://software.broadinstitute.org/morpheus).

### 2.6 High resolution single EV analysis by Imaging Flow Cytometry

For single EV analysis experiments by Imaging Flow Cytometry (IFCM), EV fractions were diluted 5-fold in PBS-HAT (DBPS supplemented with 25 μM HEPES, 0.2% human albumin and 25 μM trehalose)^15^ before usage. A volume of 5 μl was incubated with anti-human APC-conjugated CD9, CD63 and CD81 detection antibodies (all Miltenyi Biotec) at a respective final antibody concentration of 4 nM overnight. Post-staining, samples were diluted 1:2,000 in PBS-HAT buffer^15^ before acquisition on a Cellstream instrument (Amnis/Cytek) with FSC turned off, SSC laser set to 40%, and all other lasers set to 100% of the maximum power. Small EVs were defined as SSC^(low)^ by using CD63-mNeonGreen (mNG)-tagged EVs as biological reference material as described before,^21^ and regions to quantify fluorescence-positive populations were set according to unstained samples. Samples were acquired for 3 min at a flow rate of 3.66 μl/min (setting: slow) with CellStream software version 1.2.3 and analyzed with FlowJo Software version 10.5.3 (FlowJo, LLC). Dulbecco’s PBS pH 7.4 (Gibco) was used as sheath fluid. Fluorescence calibration was performed as described previously.^15^^,^^21^^,^^22^ In brief, APC MESF beads (Quantum APC MESF, Bangs Laboratories Inc., cat 823A, lot 13691) with known absolute fluorescence values for each bead population were acquired with the same settings used for EV measurements with the exception that the SSC laser was turned off, and linear regressions were performed to convert fluorescence values into APC MESF values. Flow cytometric plots using MESF unit axes were created with FlowJo v 10.5.3 (FlowJo, LLC).

### 2.7 Biofluid sampling, processing and RNA extraction

Healthy donors provided written informed consent before participation and all research was approved by the Swedish Ethical Review Authority (Diary number: 2017/912-31; 2018/952-32). Blood was collected in Sodium Citrate vacutainers (BD) and centrifuged at 2,500×*g* for 15 min within 2 h of sampling. Plasma was removed and centrifuged again at 2,500×*g* for 15 min, diluted in 0.2 μm filtered PBS and ultracentrifuged at 100,000×*g* for 90 min. EV pellets were subsequently resuspended in PBS, particles were quantified using NTA. Urine, feces, saliva, and sweat were collected in containers and 1 mL aliquots were transferred to microfuge tubes, with feces diluted 1:2 in PBS. These were then centrifuged at 2,000×*g* for 10 min and the supernatant was passed through a 0.2 μm filter to produce minimally processed, EV-containing biofluid. RNA was extracted from 1x10^8^ plasma EVs using the Maxwell Simply RNA Cells system (BioRad) or by Trizol extraction and precipitation, as previously described.^17^^,^^23^

### 2.8 RNA-sequencing

Cell culture EV RNA was extracted^17^ and precipitated as previously described^23^ by incubating 500 μl of TRI reagent (Sigma-Aldrich), adding 100 μl of chloroform and shaking vigorously. After a 15-min incubation, samples were centrifuged at 12,000×*g* for 15 min at 4 °C and 300 μl of aqueous phase was mixed with 300 μl of isopropanol, 30 μl of 3 M sodium acetate, and 1 μl of pellet paint (Merck) and incubated over night at −20 °C. The next morning, samples were centrifuged at 20,000×*g* for 30 min at 4 °C, the pellets were washed two times with 700 μl of 70% ethanol, before drying and resuspending in 15 μl of elution buffer (Qiagen). RNA concentrations were measured using Qubit RNA high-sensitivity assay (Thermo Fisher Scientific) and 2 ng was used to generate full-length complementary DNA by Smart-seq2, which uses an oligo dT primer.^24^ Fifty–base pair single-end reads were sequenced on a HiSeq3000 (Illumina), converted to fastq using bcl2fastq, adapters trimmed using Trim Galore, and the resulting reads aligned to the ENSEMBL human transcriptome GRCh37 using Tophat 2.1.1. Reads Per Kilobase per Million mapped reads (RPKM) were calculated from count matrices using edgeR package in R.^25^ For deeper coverage, RNA sequencing was repeated, reads were aligned to the ENSEMBL human transcriptome GRCh38 and visualized using Integrative Genomics Viewer (IGV, version 2.16.0).^26^, ^27^, ^28^

### 2.9 Density gradient ultracentrifugation and Smartseq3 Bioanalyzer profiles

Discontinuous iodixanol density gradients were prepared as previously described,^29^^,^^30^ by diluting a stock solution of OptiPrep™ (60% (w/v); Sigma-Aldrich) in PBS. Solutions of 40% (w/v), 20% (w/v), 10% (w/v) and 5% (w/v) were made, with the 40% and 10% solutions being prepared with a 0.45% volume and a 0.25% volume of phenol red (Sigma-Aldrich), respectively. The gradients were established by layering 6 ml of each iodixanol solution on top of each other in a 25 × 89 mm polypropylene tube (Beckman Coulter), starting with the highest density solution. Concentrated EVs were diluted in PBS to a total volume of 13.5 ml and overlaid on top of the 5% iodixanol solution. Gradients were centrifuged at 100,000×*g* in 4 °C for 16 h in a type 70 Ti fixed-angle rotor (Beckman Coulter). Eight fractions were recovered manually from the top, with fraction 1 comprising the topmost 13.5 ml PBS layer, and fraction 8 comprising the bottommost 6 ml, 40% iodixanol layer. Remaining fractions were collected as 3 ml each. All fractions were loaded separately onto 10-kDa MWCO Amicon ultracentrifugal filters (Millipore) and repeatedly washed with PBS to remove iodixanol. Samples were centrifuged at 3000×*g*, recovered in a final volume of 200 μl PBS and stored in −80 °C.^29^^,^^30^ Following RNA extraction using the Maxwell system, full-length cDNA was amplified using Smart-seq3.^31^ Amplified cDNA was then run on a Bioanalyzer 2100 High Sensitivity DNA chip and traces from each fraction are displayed.

### 2.10 RNA-seq data analysis

RNA-seq data were analyzed using R (4.3.3)^32^ with the accompanying script.^25^, ^33^, ^34^, ^35^ RPKM values of each gene were averaged over duplicates for donor samples and triplicates for cell line samples. Genes missing reads in any replicate for cell line samples were assigned 0s in all replicates. All genes were ranked according to their average RPKM value within sample and rank sums were computed for each gene across samples. Genes were ordered according to rank sums for plotting, with low rank sum corresponding to most abundantly detected genes.

### 2.11 Primer design

Primers were designed on RefSeq curated records of transcripts (NM accession numbers) using Geneious Prime Software (Dotmatics, version 2024.0.5) with a modified version of the Primer3Input algorithm (Primer3 2.3.7). Primer sequences used in this study are listed in Table 2.

**Table 1:** Top rank sum-ordered transcripts (RPKM ≥1000) shared by EVs from all 12 cell sources.

**Rank sum order**	**ENSEMBL ID**	**Gene ID**	**Gene name**	**Classification**
1	ENSG00000210082	MT-RNR2	Mitochondrially encoded 16S rRNA	Mitochondrial
2	ENSG00000203396	RP11-217O12.1	WDR45-like (WDR45L) pseudogene	Pseudogene
3	ENSG00000075624	ACTB	Actin beta	Protein-coding
4	ENSG00000251562	MALAT1	Metastasis associated Lung Adenocarcinoma transcript 1	lncRNA
5	ENSG00000087086	FTL	Ferritin light chain	Protein-coding
6	ENSG00000111640	GAPDH	Glyceraldehyde-3-phosphate Dehydrogenase	Protein-coding
7	ENSG00000236824	BCYRN1	Brain Cytoplasmic RNA 1	scRNA
8	ENSG00000205542	TMSB4X	Thymosin beta 4 X-linked	Protein-coding
9	ENSG00000115414	FN1	Fibronectin 1	Protein-coding
10	ENSG00000026025	VIM	Vimentin	Protein-coding
11	ENSG00000167995	BEST1	Bestrophin 1	Protein-coding

**Table 2:** Primer pairs used in RT-(q)PCR for validation of candidate reference transcript abundance. Listed are only primers that successfully amplified the specific PCR product in all samples.

Target gene	Primer pair	5′-3′ sequence
**TMSB4X**	1 F	AACCATGTCTGACAAACCCGATA
	1 R	AATCGTTTCTTTGGAAGGCAGTG
	2 F	TGAAGGAAGAAGTGGGGTGGAAG
	2 R	TGGGCCAGCTTGGTTTTACTCTA
**GAPDH**	1 F	TCGGAGTCAACGGATTTGGT
	1 R	TGAAGGGGTCATTGATGGCA
	2 F	CAGCCTCAAGATCATCAGCAATG
	2 R	GAGTCCTTCCACGATACCAAAGT
**ACTB**	1 F	AAAATCTGGCACCACACCTTCTA
	1 R	GGGTGTTGAAGGTCTCAAACATG
	2 F	TGTCCCCCAACTTGAGATGTATG
	2 R	TCAAGTCAGTGTACAGGTAAGCC
**VIM**	1 F	AATGGAAGAGAACTTTGCCGTTG
	1 R	GCCATCTTAACATTGAGCAGGTC
	2 F	GGACCAGCTAACCAACGACA
	2 R	TCCTCCTGCAATTTCTCCCG
**FTL**	1 F	CCGTTTTTGTGGTTAGCTCCTTC
	1 R	ATAGAAGCCCAGAGAGAGGTAGG
	2 F	TGAAGGGCCCCTTGCAAAGTAAT
	2 R	CATTTGGTCCAAGGCTTGTTAGG
**RAB13**	1 F	GCACTGTGGATATAGAGGGGAAG
	1 R	TCCACGGTAGTAGGCAGTAGTTA
	2 F	AGTGAAAGAAGGCAAGGAGGTAG
	2 R	CTGAAAACCCAGGTAAGGTCTGA
**mOx40L**	F	TCTAGACCTCGCTTTAAGTGGA
	R	GGTCTTTCGCAGGGGAACT

### 2.12 Reverse transcription

500 ng EV RNA were subjected to cDNA synthesis using the High-Capacity cDNA Reverse Transcription Kit (Thermo Fisher Scientific) and a 15-mer oligo-dT primer according to the manufacturer’s protocol. Briefly, 10 μl of a 2X RT master mix consisting of ddH_2_O, 2X RT buffer, 8 mM dNTPs, 20 μM oligo-dT primer and 50 U MultiScribe Reverse Transcriptase was mixed with 10 μl RNA and incubated for 10 min at 25 °C followed by 2 h at 37 °C in a thermocycler. The reaction was stopped by heating to 85 °C for 5 min cDNA samples were diluted 1:5 with ddH_2_O to a final volume of 100 μl.

### 2.13 Endpoint PCR

Endpoint PCR was performed on cDNA transcribed from EV RNA using the Qiagen HotStarTaq Plus MasterMix according to the manufacturer’s instructions. Briefly, each reaction was set up using a final concentration of 1X MasterMix, 0.5 μM forward primer, 0.5 μM reverse primer, 1X CoralLoad PCR Buffer, 1 μl cDNA template and ddH_2_O to a total volume of 15 μl per reaction. Cycling conditions were as follows: initial denaturation 95 °C for 5 min, then 40 cycles of 94 °C for 30 s, 62 °C for 30 s, and 72 °C for 1 min, followed by final extension at 72 °C for 10 min. PCR products were analyzed by gel electrophoresis on 2% agarose gels prepared with 2% (w/v) SeaKem LE Agarose (Lonza) in 1X Tris-Borate-EDTA (TBE) running buffer.

### 2.14 Quantitative PCR

Quantitative real-time PCR (qPCR) was performed using the PowerUp SYBR Green Master Mix (Thermo Fisher Scientific), a pre-formulated universal 2X master mix containing Dual-Lock Taq DNA polymerase for real-time PCR. Briefly, a 10 μl qPCR reaction was set up using 5 μl 2X master mix, forward primer at 200 nM final concentration, reverse primer at 200 nM final concentration, 1 μl cDNA and ddH_2_O. Samples and standards were measured in triplicates using the CFX96 Touch Real-Time PCR Detection System (BioRad). Cycling conditions were used as follows: 1. 50 °C for 2 min, 2. 95 °C for 2 min, 3. 95 °C for 15 s, 4. 62 °C for 1 min + plate read, 5. go to 3 for 39 more times, 6. 95 °C for 15 s, 7. melt curve from 60 °C to 95 °C (increment 0.5 °C) for 5 s + plate read.

### 2.15 Digital PCR

Digital PCR (dPCR) was performed using the partition-based Qiagen QIAcuity dPCR system according to the manufacturer’s recommendations. The pre-formulated universal 3X EvaGreen PCR master mix is optimized for microfluidic use in the QIAcuity Nanoplates and contains the QuantiNova Taq DNA polymerase, EvaGreen as intercalating dsDNA dye, and an optimized reference dye. Briefly, a 12 μl qPCR reaction was set up using 4 μl 3X master mix, forward primer at 0.4 μM final concentration, reverse primer at 0.4 μM final concentration, 1.2 μl cDNA and ddH_2_O. 11 μl of reaction mixture were transferred to the respective well in a QIAcuity 8.5k 24-well Nanoplate and measured using the QIAcuity Four dPCR system. Cycling conditions were used as follows: 1. 95 °C for 2 min, 2. 95 °C for 15 s, 3. 62 °C for 15 s, 4. 72 °C for 15 s, 5. go to 2 for 39 more times, 6. 40 °C for 5 min, 7. Imaging Green channel at 400 ms exposure with Gain 3. Data was analyzed using QIAcuity Software Suite 2.5.0.1.

### 2.16 Additional software and open-access databases

Proof-of-concept normalizations using RPKM and Cq values, as well as Spearman rank correlation coefficient r and linear regression analysis were computed and visualized using GraphPad Prism Software (version 10.1.2). For single transcript analysis public databases for genome annotations were consulted: UCSC Genome Browser (NIH, http://genome.ucsc.eu)^36^ and Ensembl Genome Browser (EMBL-EBI, http://ensembl.org), browser releases 111 and 112. Illustrations were prepared using BioRender and publication licenses are available upon request.

## 3 Results

### 3.1 Identification of seven common mRNA transcripts associated with EVs from 12 different cell sources

To identify potential EV mRNA reference transcripts applicable across various cell sources, we analyzed an RNA-seq dataset prepared from 12 different cell line EVs previously published by our group (Fig. 1A).^37^ As reported, EVs were isolated by differential centrifugation, tangential flow filtration (TFF) and ultrafiltration, and validated by Nanoparticle Tracking Analysis (NTA) as well as multiplex bead-based EV flow cytometry (data reproduced from Hagey et al. (2023) and depicted in Supplementary Figure S 1).^19^^,^^20^^,^^37^ Based on their tissue of origin, the EV cell sources were categorized into five groups: (1) immune cell EVs from THP-1 and JURKAT cells, (2) EVs from cells of in-utero origin, including human amniotic epithelium (HAEC) and CEVEC’s Amniocyte Production (CAP) amniotic fluid cells, (3) epithelial cell-derived EVs from HUVEC and PANC-1 cells, (4) embryonic kidney cell-derived EVs from HEK293-T and HEK293-FreeStyle (FS) cells, and (5) EVs from mesenchymal or fibroblast origins, including Wharton's Jelly mesenchymal stem cells (WJ-MSC), bone marrow-derived mesenchymal stromal cells (BM-MSC), umbilical cord-derived mesenchymal stromal cells (CB-MSC), and fibroblasts (BJ-5ta).^37^ Since appropriate reference transcripts for normalization are ideally highly and consistently abundant across biological samples,^5^^,^^6^ transcripts with no expression (RPKM = 0) in any of the triplicates for each EV sample were excluded. To identify common transcripts in all 12 EV samples, the top 1000 most abundant transcripts in each EV sample were analyzed further (Fig. 1B). This identified 58 transcripts shared by all 12 EV samples, unique transcripts for each EV sample, as well as transcripts partly shared between some but not all EVs (Fig. 1B). Next, all genes were rank sum-ordered according to the average RPKM value per sample and the top 50 of highly abundant transcripts were selected for detailed analysis (Supplementary Figure S 2A). Further selection was achieved by applying a cutoff at 1000 RPKM based on the overall RPKM distribution, i.e. excluding all transcripts with RPKM <1000, to select transcripts with robustly high abundance only (Supplementary Figure S 2B). This analysis reduced the list of commonly shared transcripts across all 12 samples to eleven genes (Fig. 1C and Table 1). Focusing on protein-coding transcripts, we subsequently excluded non-protein-coding and mitochondrial transcripts as well as pseudogenes, ultimately identifying seven final candidate reference transcripts: ACTB, FTL, GAPDH, TMSB4X, FN1, VIM, and BEST1.

### 3.2 Validation of five candidate reference genes by RT-qPCR

Despite the widespread use of deep sequencing and high-throughput analysis methods, many research laboratories lack the resources to perform these techniques routinely. RT-qPCR remains the most commonly used method for mRNA quantification due to its accessibility, sensitivity, specificity, and broad dynamic range. Therefore, we next performed RT-qPCR to validate the seven robustly expressed transcripts we identified by RNA-seq to facilitate widespread use of these reference genes in the EV community.

To accomplish this, independent batches of oligo-dT primed cDNA were prepared from EV-derived RNA from each cell source and used as template for PCR and qPCR. Additionally, qPCR was performed on the library cDNA templates previously used for RNAseq analysis. To ensure accurate primer design, the genomic alignments for each of the seven candidate transcripts were examined for sequence coverage, sequencing depth, and consistency across all samples. For FN1, which is annotated with a primary transcript exceeding 75 kb in length, only one sample (BM-MSC) showed full coverage of the annotated transcript, while the other 11 EV samples displayed only irregular read coverage across the FN1 gene sequence (Supplementary Figure S 3A). However, a common peak of less than 100 bp mapped to an intronic region was identified (Supplementary Figure S 3A). Similarly, for BEST1, the only common read coverage across most EV samples (11 out of 12) was a region of less than 100 bp in a RefSeq-annotated exon, while other common reads were mapped to intronic regions (Supplementary Figure S 3B). For both FN1 and BEST 1, regions of shared coverage over all samples were either not in annotated coding regions or too short for reliable qPCR amplification. This led us to conclude that FN1 and BEST1 transcripts are unsuitable as mRNA standards for analysis in EVs and they were therefore excluded from further analysis.

**Fig. 2:**
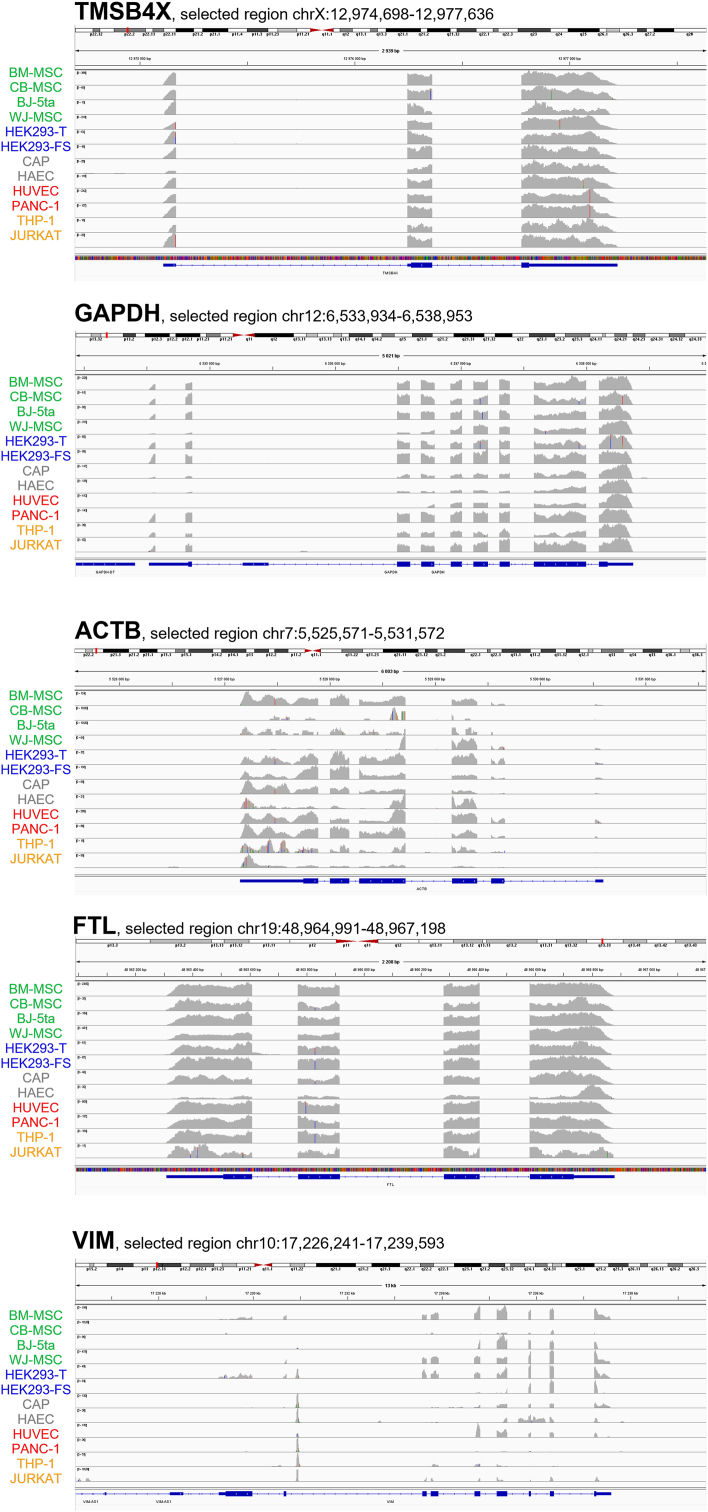
RNA-seq read coverage per sample of the five candidate reference transcripts also validated by RT-qPCR. Selected genomic regions according to GRCh38 are shown per gene, with RefSeq annotated gene structure at the bottom of each panel. Aligned reads were mapped to the annotated gene for each EV sample and coverage is shown in grey.

Since TMSB4X, GAPDH, ACTB, FTL, and VIM showed full length sequencing coverage across all samples (Fig. 2), at least two RT-qPCR primer pairs were designed against each transcript (Table 2). Primer performance was validated using conventional RT-PCR with freshly prepared oligo-dT primed cDNA as template (Supplementary Figure S 4A). P rimer pairs that produced clear amplicons in all samples were selected for further qPCR analysis. All five transcripts were successfully amplified, with mean Cq values ranging from 22.9 to 36.14 across all samples (Supplementary Figure S 4B). To confirm these results, RT-qPCR was also performed using the Smart Seq2 library cDNA templates previously analyzed by RNA sequencing. All five transcripts (VIM, TMSB4X, ACTB, GAPDH, and FTL) were amplified in all 12 samples using these primer pairs, with mean Cq values ranging from 11.22 to 31.51 across all samples (Supplementary Figure S 4B).

### 3.3 Stability assessment of the candidate reference transcripts

**Fig. 3:**
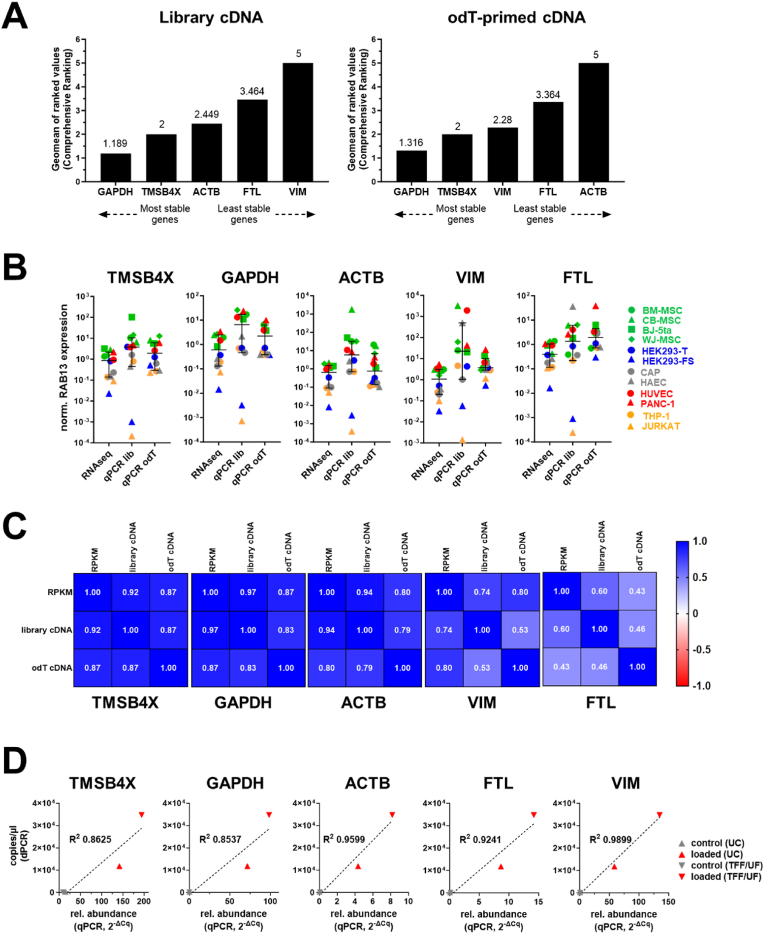
Suitability of candidate reference genes for normalization. **(A)** Stability evaluation for each of the five candidate reference transcripts by the online tool RefFinder using RT-qPCR data obtained from sequencing library cDNA samples (left) or freshly prepared oligo dT-primed cDNA. Shown are results from the comprehensive ranking incorporating four different analysis algorithms. **(B)** Proof-of-concept normalization of RAB13 expression to each respective candidate reference transcript using RPKM values from the RNA-seq dataset and Cq values from two independent RT-qPCR assays on either library cDNA or freshly prepared oligo dT-primed cDNA. Shown are normalized values for each EV sample, with median and 95% CI. **(C)** To assess the suitability for normalization using RNA-seq or RT-qPCR data, respective normalized RAB13 expression values were ranked and compared by computing the Spearman correlation coefficient ρ for each candidate reference gene. ρ = 1: perfect association of rank, ρ = 0: no correlation of rank, ρ = −1, perfect negative association of rank. odT – oligo dT-primed. **(D)** To further validate the use reference transcripts in RT-qPCR, the relative abundance of a target mRNA measured in RT-qPCR was tested in comparison to absolute copy numbers determined by dPCR. Murine Ox40L mRNA was actively (loaded) or passively loaded (control) into EVs as published,^16^ and EVs were isolated by two methods (UC or TFF/UF). Absolute copies per μl cDNA were plotted against the 2^-ΔCq^ value of mOx40L expression over the indicated reference transcript (ΔCq = Cq_mOx40L_ – Cq_reference_). The linear correlation of both data sets was assessed by a linear regression curve analysis, with R^2^ = 1 corresponding to a perfect curve fit. R^2^ values for each analysis are indicated in the respective diagram. UC – Ultracentrifugation, TFF – Tangential Flow Filtration, UF – Ultrafiltration.

To assess the stability of these five transcripts, we used RefFinder^38^^,^^39^ to analyze the RT-qPCR datasets from these two independent reverse transcription reactions of the same EV samples. RefFinder is an online tool that integrates four major computational algorithms into a comprehensive ranking assessing reference gene stability using RT-qPCR datasets: the Comparative Delta Ct method,^40^ BestKeeper,^41^ Normfinder,^42^ and GeNorm.^43^ The Comparative Delta Ct method ranks transcript stability based on the standard deviation of input Cq values.^40^ BestKeeper calculates an index of stability by analyzing amplification efficiencies and Cq values, followed by pairwise correlation analysis.^41^ NormFinder evaluates variation within and between sample groups using a mathematical model of gene expression.^42^ GeNorm assesses transcript expression stability by calculating standard deviations and progressively excludes the least stable genes until the two most stable remain.^43^ RefFinder analysis for both RT-qPCR datasets classified GAPDH and TMSB4X as the two most stable genes according to the comprehensive ranking (Fig. 3A). The remaining three genes (FTL, ACTB, and VIM) were ranked as the third to fifth most stable, with varying order depending on the dataset and ranking algorithm (Fig. 3A).

### 3.4 Proof-of-concept normalization of target transcript

To evaluate the robustness of the five top-ranked candidate reference genes, we aimed to conduct a proof-of-concept normalization using an mRNA transcript with consistent, but highly variable expression over all 12 samples. To this end, we selected RAB13 as gene of interest from the RNA-seq dataset based on its common expression in all 12 samples, but wide variance in RPKM value rank as compared the potential reference genes (Supplementary Figure S 5A). RAB13 encodes for a transcript of 1.3 kb in length, which was consistently present in all 12 samples at varying expression levels (Supplementary Figure S 5B, C). We proceeded to validate the presence of RAB13 by performing RT-qPCR on cDNA templates from both the original RNA-seq libraries and newly synthesized oligo-dT primed cDNA (Supplementary Figure S 5C). To test the suitability for normalization of the five candidate reference transcripts, RAB13 RPKM values were normalized against the RPKM values of TMSB4X, GAPDH, ACTB, VIM, and FTL, respectively, to obtain relative RAB13 expression levels for each EV sample (Fig. 3B). Similarly, normalization of the RAB13 RT-qPCR data was performed using the Delta Ct method^40^ for both RT-qPCR datasets, derived from either the library cDNA or the newly synthesized oligo dT-primed cDNA templates (Fig. 3B).

To assess the correlation between RNA-seq and RT-qPCR as normalization methods, the normalized RAB13 values from the three respective datasets were compared across all 12 samples. For each reference transcript, the normalized RAB13 values were ranked from 1 to 12, with 12 representing the highest expression. We then compared the rank orders across the three methods and calculated the Spearman rank correlation coefficient (ρ) to determine the degree of correlation. The strongest correlation between the three methods was observed for TMSB4X and GAPDH, with ρ values ranging from 0.87 to 0.92 for TMSB4X and 0.83 to 0.97 for GAPDH (Fig. 3C). ACTB followed with ρ values between 0.79 and 0.94. VIM showed moderate correlation (ρ values between 0.53 and 0.74), while FTL had the lowest correlation, with ρ values ranging from 0.43 to 0.6 (Fig. 3C). This analysis confirmed the suitability of TMSB4X, GAPDH, ACTB, VIM and FTL as reference transcripts for mRNA normalization in RT-qPCR across a broad range of EV source cells.

### 3.5 Benchmarking of reference transcript normalization to digital PCR

The objective of identifying reference transcripts for qPCR normalization is to improve accessibility to standardized measurements when absolute quantification is not available. Digital PCR (dPCR) achieves absolute quantification by massive parallel measurement after diluting input material to single molecule concentrations. To produce input material with known enrichment of a specific transcript for benchmarking, we used a recently developed mRNA loading strategy: By transfecting the sequence-specific RNA-binding protein PUFe fused to CD63 into cells stably expressing murine Ox40L (mOx40L) mRNA with the compatible 3′end PUFe-binding sequence, we could control the abundance of this transcript in EVs.^16^ Thus, to benchmark our reference transcripts to dPCR, we compared the quantification of mOx40L in two independently produced biological replicate preparations of EVs, one purified by UC and the other purified by TFF/UF, both in the presence (loaded) or absence (control) of CD63-PUFe (Fig. 3D). This analysis revealed a strong correlation between transcript copy numbers determined by dPCR and the relative RT-qPCR quantifications achieved by each of our reference genes, with higher mOx40L mRNA loading consistently observed in the biological replicate isolated by TFF/UF than in the sample where EVs were purified by UC. Thus, normalization to any of the five candidate reference genes reflected the true enrichment of target mRNA over control, while also showing the natural variation in loading between the two independently produced EV preparations.

### 3.6 Analysis of reference transcript distribution by density gradient centrifugation

**Fig. 4:**
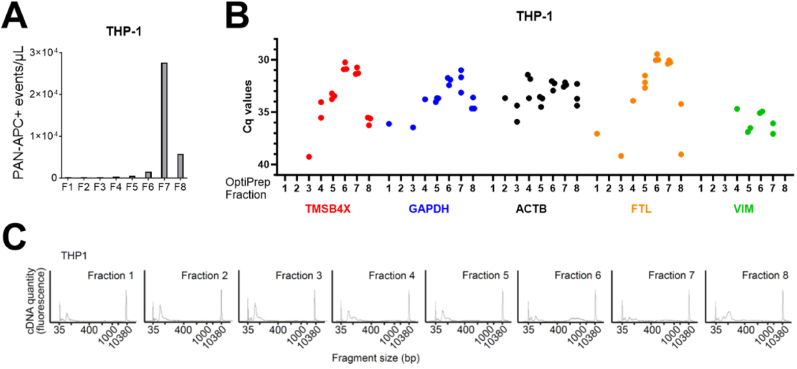
EV mRNA transcripts are highly abundant in OptiPrep density gradient-purified EVs. **(A)** EV flow cytometry assay detecting the characteristic tetraspanin protein signature (“PAN-APC” antibody mix: CD9, CD63, and CD81-antibodies labelled with APC) in all 8 fractions harvested by OptiPrep density gradient centrifugation from THP-1 EVs. The majority of all events was detected in fraction 7. **(B)** RT-qPCR analysis of the five candidate reference transcripts, TMSB4X, GAPDH, ACTB, FTL, and VIM, in fractions 1–8 harvested from THP-1 EVs. Abundance peaks (lowest Cq value) were consistently observed in fractions 6 and7. **(C)** Full-length Smart-seq3 cDNA profiles run on a Bioanalyzer 2100 High Sensitivity chip.

Since our reference genes were identified in mRNA-sequencing data from EVs isolated by TFF/UF, it was important to confirm the association of these transcripts with EVs isolated by more stringent methods. Thus, we performed density gradient centrifugation on TFF/UF isolated EVs from THP-1 and HEK293-FS cells. We then used Imaging Flow Cytometry to determine the distribution of CD9, CD63 and CD81-bearing EVs throughout the gradient fractions and found these to be detectable between fractions 4–8, with a peak in fraction 7 for THP-1, and fraction 6 for HEK293-FS-derived EVs (Fig. 4A, Supplementary Figure S 6A-B). RNA was then isolated from each fraction and RT-qPCR targeting each of our five candidate reference genes was performed. For THP-1-derived EVs, all of the reference transcripts were most abundant between gradient fractions 4–7, with the majority of them showing clear peaks of expression in fractions 6 and 7 (Fig. 4B). To determine if this distribution of transcripts could be extended to full-length mRNA more generally in our EV preparations, we performed Smart-seq3 and visualized the resulting cDNA profiles from each gradient fraction by capillary electrophoresis. The greatest amounts of full-length cDNA were detected in fraction 6, followed by fraction 7 (Fig. 4C). HEK293-FS-derived EVs, their associated reference transcripts and full-length mRNA were more broadly distributed between gradient fractions 4–8, but also followed a pattern whereby transcripts were most abundant in the gradient fractions directly preceding those of peak EV concentrations (Supplementary Figures S 6B-D).

### 3.7 Reference transcript abundance in biofluid-derived EVs

**Fig. 5:**
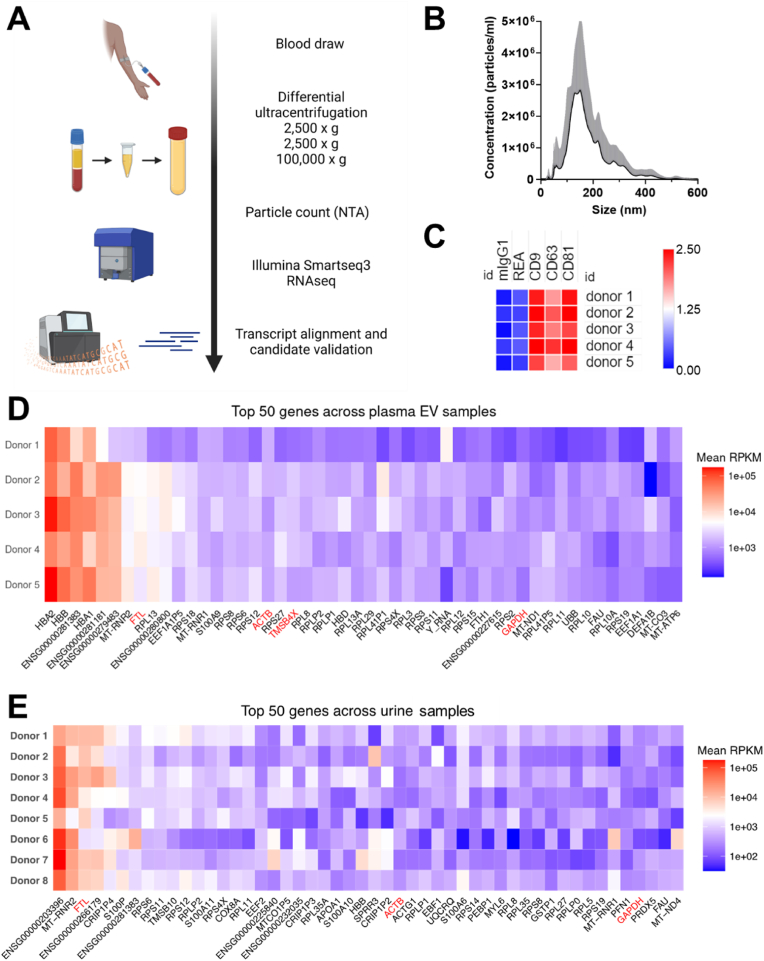
Four reference transcripts are highly abundant in plasma EV mRNA. **(A)** Workflow schematic depicting the isolation of plasma EVs from healthy donors, followed by NTA and RNA-seq analysis. Figure created using BioRender. **(B)** NTA profiles of plasma EVs isolated from 5 healthy donors. **(C)** Quantification of abundant tetraspanin EV markers by multiplex bead-based EV flow cytometry. Heatmap shows log10-fold change over control (beads only + antibody) **(D)** Four previously identified reference transcripts, FTL, ACTB, TMSB4X, and GAPDH (all marked in red font), are detected among the rank-sum ordered top 50 transcripts in plasma EVs from 5 healthy donors. **(E)** Three of the reference transcripts, FTL, ACTB and GAPDH (all marked in red font), are detected among the rank-sum ordered top 50 transcripts in minimally processed urine from eight healthy donors.

To further extend the applicability of the identified reference transcripts, their abundance in EVs derived from human blood plasma was assessed. This analysis evaluated the suitability of these reference genes for normalization of expression data obtained from EVs isolated from biological fluids, which are of significant interest in clinical and diagnostic research. To this end, blood plasma-derived EVs, collected from platelet-poor plasma of five healthy donors, were isolated using differential ultracentrifugation (Fig. 5A). The particle sizes were measured by NTA at an average modal size of 128.4 ± 25.2 nm (Fig. 5B) and the presence of EVs was validated by multiplex bead-based EV flow cytometry analyzing the abundance of tetraspanin markers CD9, CD63, and CD81 commonly present on EVs (Fig. 5C). Furthermore, we analyzed minimally processed samples of urine, feces, saliva, and sweat from eight donors to understand how broadly applicable these reference transcripts may be (Supplementary Fig. S7).

For RNA-seq, oligo-dT primed cDNA libraries were prepared from the total RNA of these EVs and subjected to high-throughput Illumina sequencing. As with the previous dataset, we rank sum-ordered the transcripts based on their average RPKM values per sample, identifying the top 50 most highly abundant genes across all five plasma EV samples (Fig. 5D). Interestingly, among the top-ranked genes, a predominance of transcripts associated with hemoglobin biosynthesis was observed. Despite this, four out of the five candidate reference genes, FTL, ACTB, TMSB4X, and GAPDH were listed among the 50 top-ranked transcripts in the plasma-derived EVs (Fig. 5D). When looking at minimally processed biological fluids, FTL and ACTB were among the 50 most abundant transcripts in all but sweat, where none of our reference genes were represented (Fig. 5E and Supplementary Fig. S7B-D). In addition, GAPDH was amongst the most highly represented genes in urine (Fig. 5E) and TMSB4X was one of the 50 most abundant extracellular mRNAs in feces (Supplementary Fig. S7C). This suggests that these genes are expressed at consistently high levels not only in cell culture-derived EVs, but also in EVs originating from most complex biological fluids including blood and urine.

## 4 Discussion and conclusions

Normalization of RT-qPCR data from EV RNA preparations has been a matter of investigation since the discovery of RNA cargo molecules in EVs almost two decades ago.^3^ However, due to the heterogeneity of EV subpopulation and the currently limited knowledge of RNA cargo in EVs, only a few studies have successfully postulated RNA transcripts that could be used as normalization references. As EVs are most often investigated in relation to small RNAs,^12^ small RNA reference transcripts for the normalization of RT-qPCR data have been established for EVs of numerous cellular origins.^44^ Due to the much lower content of longer RNA transcripts,^12^^,^^45^ the identification of reference mRNA transcripts has proven more difficult. One of the few studies investigating this identified GAPDH, YWHAZ, and UBC as the most stable reference mRNA transcripts in EVs derived from cell lines of liver or breast cancer origin.^7^ In that study, the objective was to test eight commonly used cellular reference genes in EVs derived from these tissues, and validate them for normalization of EV-derived mRNA quantification by RT-qPCR.^7^ A more unbiased approach was published recently, where four genes, SNRPG, NOP10, TOMM7, and OST4, were identified as specifically enriched in RNA-seq data obtained from EVs isolated by density gradient ultracentrifugation as opposed to other purification methods.^8^ Their presence was subsequently confirmed by RT-qPCR in EVs from various cell lines, at different experimental conditions, biological fluid-derived EVs, and reference EV preparations.^8^ Despite these studies, there are no general recommendations on how to normalize EV RNA quantification by RT-qPCR apart from performing careful selection of the analysis method or settling on absolute mRNA quantification using either qPCR standard curves or digital PCR approaches.^10^^,^^11^ This lack of consensus mirrors the experimental difficulties the field encounters, and illustrates current difficulties in proving accuracy, robustness, and universal applicability of the postulated reference transcripts.

In our study we aimed at providing a comprehensive and unbiased top-down approach to the identification of global mRNA reference transcripts widely applicable for EVs prepared by scalable methods. We attempted this by analyzing EV RNA-seq data derived from a multitude of cell sources to pinpoint transcripts that are consistently and highly enriched across a wide range of EV populations. Through rigorous evaluation of transcript abundance for all samples, we narrowed this list down to seven potential reference genes, and subsequently validated five of these, TMSB4X, GAPDH, ACTB, VIM, and FTL, using RT-qPCR. Among those, TMSB4X, GAPDH, ACTB, and FTL were also identified within the top-ranked transcripts in blood plasma-derived EVs and at least two of the minimally processed biofluids we analyzed. Given the strong correlation between RNA-seq and RT-qPCR results demonstrated for cell culture-derived EVs, TMSB4X, GAPDH, ACTB, and FTL could potentially serve as good reference transcripts for the normalization of RT-qPCR data from biological fluid-derived EVs as well. These will be necessary to extend EV research into clinical settings, where the reliable normalization of EV RT-qPCR data is crucial for both diagnostic and therapeutic applications. Although FTL and ACTB were amongst the 50 most abundant transcripts in the other biofluids we studied, it was interesting that none of our reference genes were robustly found in sweat. Although these were amongst the top 500 most abundant transcripts in sweat, this illustrates the underlying fact that truly universal standards do not exist for the full diversity of EV biology and should always be confirmed for the system being investigated.

The heterogeneity of vesicle populations substantially influences their overall RNA content, RNA species composition, and, therefore, their most appropriate reference transcripts. Common EV isolation techniques include density gradient or differential ultracentrifugation, size-exclusion chromatography, ultrafiltration, and immunoaffinity capture. Each of these methods may enrich for different subpopulations of EVs, potentially leading to variability in RNA profiles.^30^^,^^46^ For example, simple filtration co-isolates all non-EV particles that pass the filter.^46^^,^^47^ On the other hand, immunoaffinity capture targets specific EV surface markers, which could result in a more homogenous EV population but excludes EVs lacking the targeted marker, thus influencing the overall RNA landscape.^12^ Thus, it was important that we confirmed the overlap between EV and full-length reference transcript abundance following density gradient centrifugation. Interestingly, the peak in mRNA abundance was generally one fraction before that of CD9, CD63, and CD81, suggesting this may be most abundant in a larger than average population of EVs. This demonstrates the complexity of identifying reference targets for different cargo in diverse and poorly understood biological nanoparticle mixtures.

As EVs are postulated to mirror the macromolecular content of their cell of origin, the RNA cargo composition in EVs largely depends on their tissue of origin.^48^ In this study we included a broad, although non-exhaustive, selection of EV sources and identified the proposed reference transcripts by a top-down approach including high abundance in all cell types. Thus, the validity of the reference genes for EVs identified in our study is most likely not restricted to certain tissues only, as previously reported for others.^7^ In fact, the high abundance, stability, and low variation of these transcripts in both RNA-seq and RT-qPCR datasets over all 12 samples and most biofluids suggests that these transcripts are broadly applicable. Nevertheless, appropriate validation of their applicability in a specific experimental setting is always recommended.

Our findings contribute significantly to the current literature, which increasingly underscores the importance of validated reference genes in EV research. Traditional housekeeping genes, often used in cellular RNA studies, may not be suitable for EVs due to their distinct RNA composition and the influence of isolation and processing methods. By focusing specifically on EV-derived RNA and applying stringent criteria for reference gene selection, our study addresses this gap and provides a set of reference transcripts better suited to the unique characteristics of EV research. The subsequent validation of these transcripts using RT-qPCR in a broader experimental context further highlights their utility in routine laboratory settings, where access to high-throughput sequencing technologies may be limited.

Analysis of the alignments show, that all four of the final candidate transcripts, TMSB4X, GAPDH, ACTB, and FTL, were detected in full length within EV-derived mRNA. In fact, GAPDH and ACTB have previously been reported as the most abundant full-length mRNA transcripts in EV RNA originating from cell types other than those analyzed in our study.^45^^,^^49^ It is therefore encouraging to observe similar results in our dataset and warrants the speculation that the mRNA transcripts might have a function in cell-cell communication and might be preferentially sorted into EVs during biogenesis. The true biological functions and mechanistic aspects of the sorting of these reference transcripts into EVs, however, remain to be fully elucidated.

In conclusion, this study advances the field of EV research by providing a set of rigorously validated reference transcripts for mRNA normalization. The identification of TMSB4X, GAPDH, ACTB, and FTL as stable reference genes offers a reliable foundation for quantifying mRNA in EVs across a broad range of cell sources and biofluids, facilitating more accurate and reproducible studies. As the field continues to evolve, it will be essential to validate these reference genes across various EV isolation methods and broader EV populations to ensure their applicability across different experimental contexts. Standardizing reference gene selection is crucial for advancing our understanding of EV biology and enhancing their potential as biomarkers in clinical and translational research. This work underscores the importance of methodological rigor in EV studies and provides a pathway toward more accurate and reliable EV mRNA analyses, ultimately contributing to the broader field of EV-based diagnostics and therapeutics.

## CRediT authorship contribution statement

**Antje M. Zickler:** Writing – review & editing, Writing – original draft, Visualization, Validation, Project administration, Methodology, Investigation, Formal analysis, Data curation, Conceptualization. **Radosław Grochowski:** Writing – review & editing, Visualization, Validation, Software, Investigation, Formal analysis, Data curation. **André Görgens:** Writing – review & editing, Visualization, Methodology, Investigation, Funding acquisition, Formal analysis. **Erik Bäcklin:** Investigation, Methodology, Writing – review & editing. **Maximilian Kordes:** Resources, Investigation. **J.-Matthias Löhr:** Resources, Investigation. **Joel Z. Nordin:** Writing – review & editing, Supervision, Funding acquisition. **Samir EL Andaloussi:** Writing – review & editing, Supervision, Project administration, Funding acquisition, Conceptualization. **Daniel W. Hagey:** Writing – review & editing, Software, Resources, Project administration, Methodology, Investigation, Funding acquisition, Data curation.

## Data availability

RNA-seq data and code used for its analysis is available at https://github.com/radee2k/Identification-of-robust-and-abundant-EV-reference-mRNA-transcripts-from-diverse-biological-sources.

## Funding information

• European Research Council (ERC) under the European Union’s Horizon 2020 research and innovation programme (DELIVER, grant agreement No 101001374) (S.E.A.)
• European Union’s Horizon 2020 research and innovation programme (EXPERT, grant agreement No 825828) (S.E.A.)
• Swedish foundation of Strategic Research FormulaEx, SM19-0007 (S.E.A.)
• Cancerfonden project grant 21 1762 Pj 01 H (S E.A.), 21 0378 PT (D.W.H.)
• Barncancerfonden TJ2022-0076 (D.W.H.)
• Ming Wai Lau Foundation (S.E.A)
• Åke Wiberg Foundation (D.W.H.)
• Alex and Eva Wallströms Foundation (D.W.H.)
• 10.13039/501100004359Swedish Research Council grant 4–258/2021 (S.E.A.)
• 10.13039/501100004359Swedish Research Council grant 2021-02407 (J.N.)
• CIMED junior investigator grant (J.N. & D.W.H.)
• 10.13039/501100004047Karolinska Institutet10.13039/100031212Network Medicine Alliance Grant (A.G.)
• International Society for Advancement of Cytometry (ISAC) Marylou Ingram Scholarship 2019–2024 (A.G.)

## Declaration of competing interest

These authors declare the following potential conflict of interests:

• S.E.A. is a co-founder, consultant, and stakeholder of Evox Therapeutics Ltd.
• J.Z.N. and A.G. are consultants and stakeholders of Evox Therapeutics Ltd.

All other authors (A.M.Z., R.G., E.B., M.K., J.-M.L., D.W.H.) declare no potential conflict of interest.
