# Supplementary material for: Identification of robust and abundant reference transcripts for EV mRNA cargo normalization
Source: Extracell Vesicle. 2025 Jun;5:None. doi: 10.1016/j.vesic.2025.100065 (PMC12199199; doi:10.1016/j.vesic.2025.100065)
Supplement: Multimedia component 1 [file mmc1.docx]

**Supplementary Data**


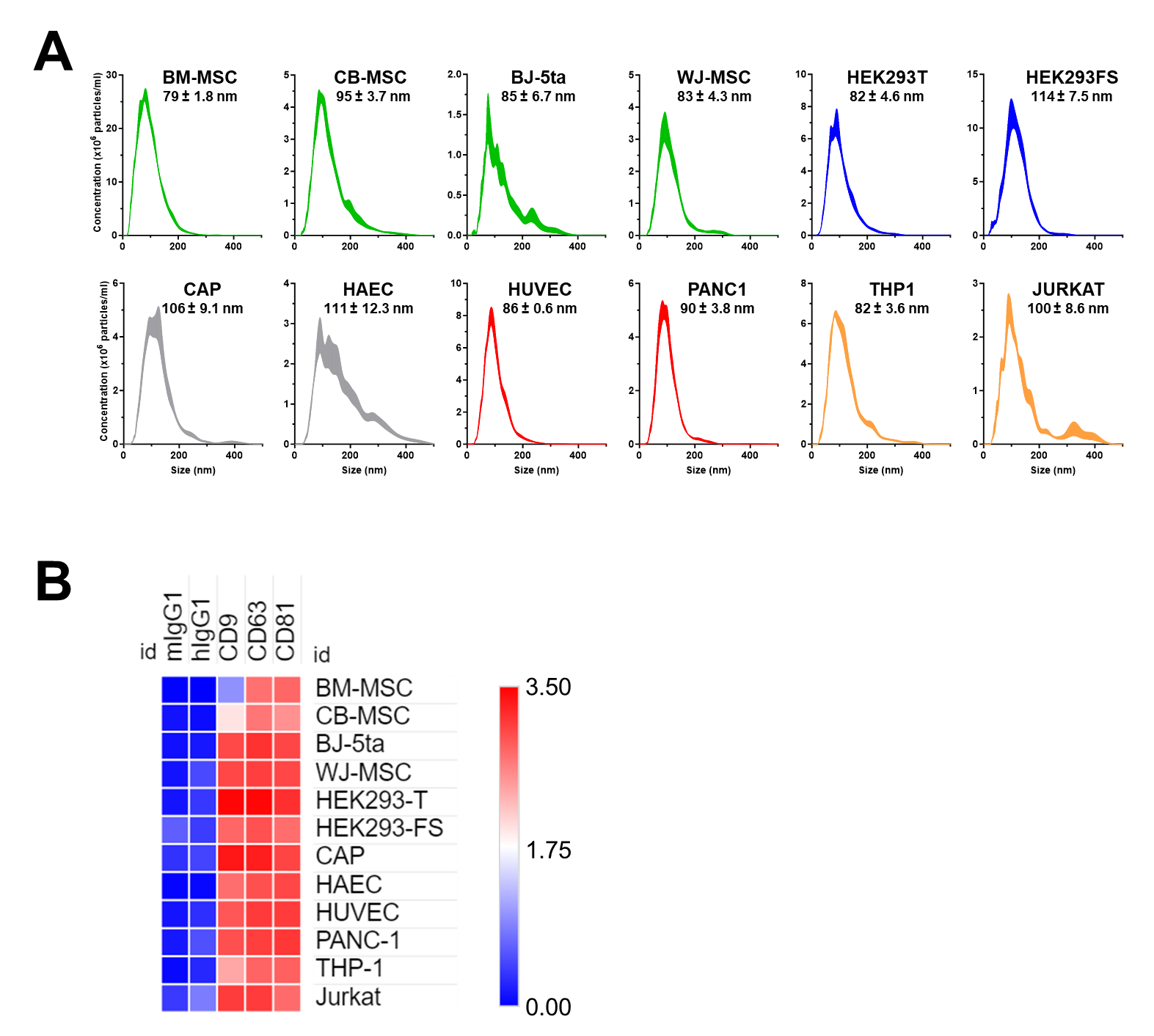


**Supplementary Figure S 1**: Characterizaton of EVs from 12 cell sources, reproduced and adapted from Hagey et al., Sci Adv, 2023.^[38]^ (A) Particle size distributions measured by NTA and modal particle size per EV type as indicated by the source cell line. (B) Quantification of abundant tetraspanin EV markers by multiplex bead-based EV flow cytometry. Heatmap shows log10-fold change over control (beads only + antibody).


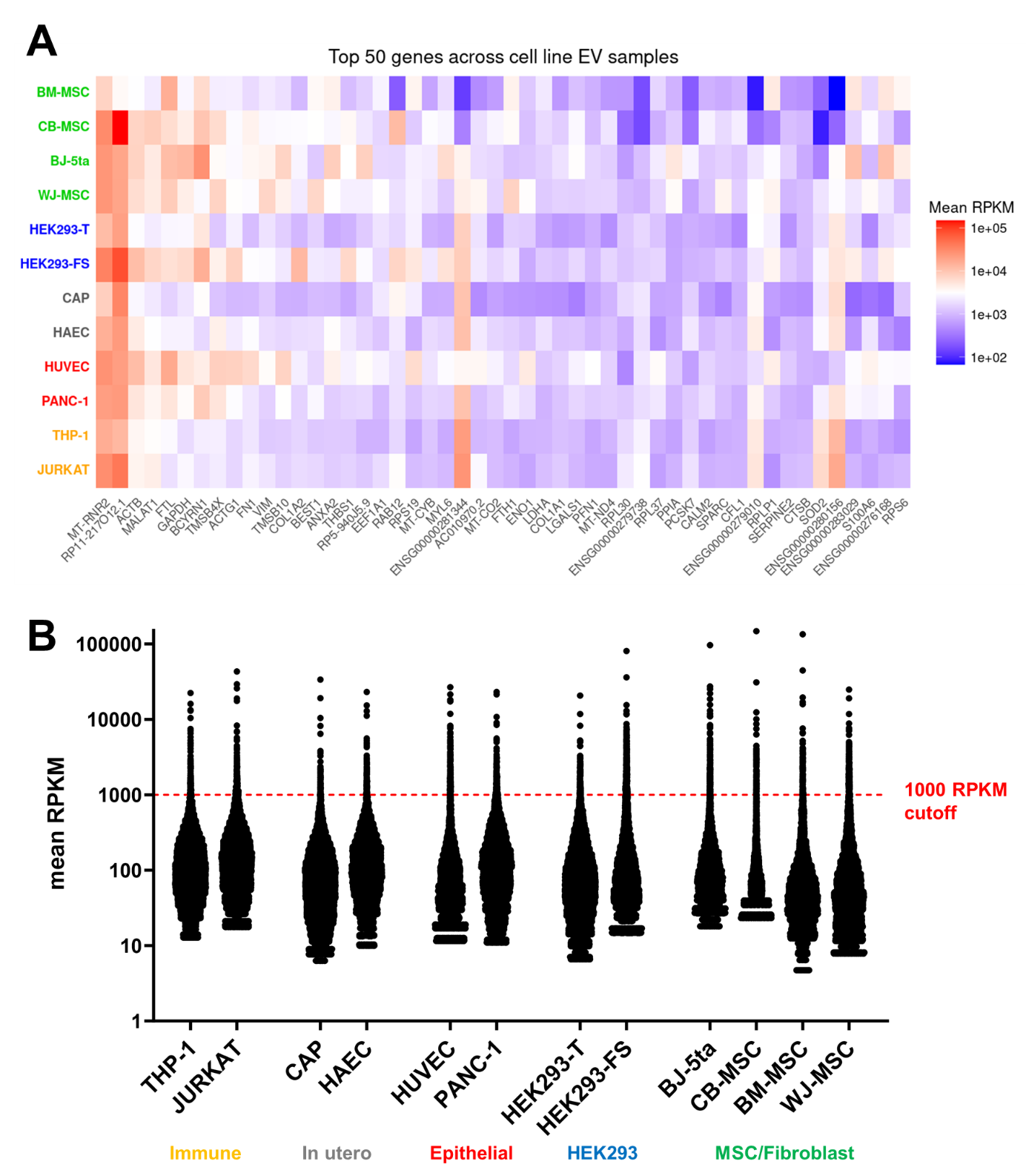


**Supplementary Figure S 2**: Top-ranked transcripts in EVs from 12 cell sources. **(A)** Rank-sum ordered top 50 transcripts. **(B)** RPKM value distribution per EV type with the level of RPKM = 1000 cut-off implemented for analysis of highly abundant transcript only.


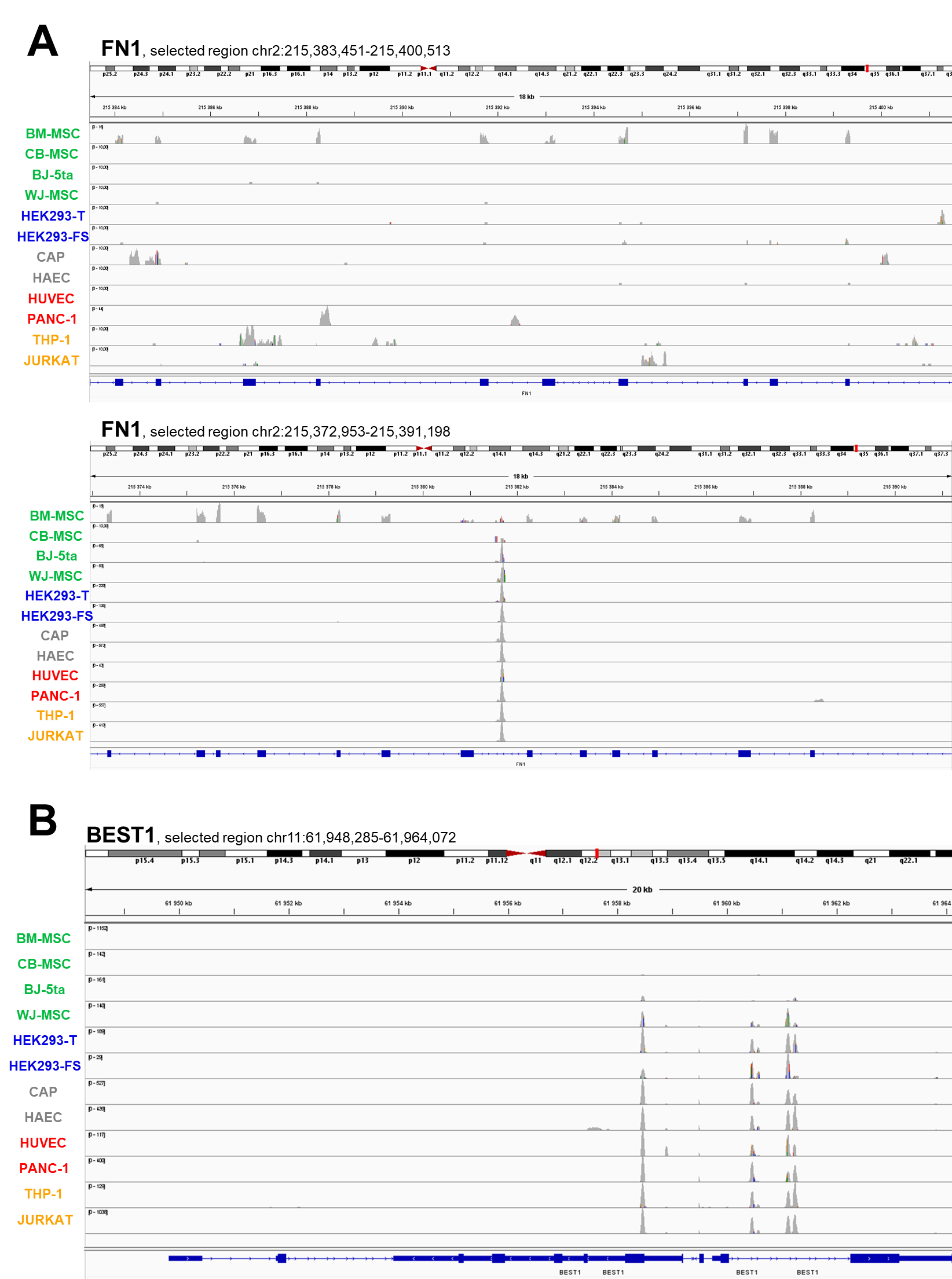


**Supplementary Figure S 3**: Representative RNA-seq read coverage visualizations per sample of the two excluded candidate reference transcripts **(A)** FN1 and **(B)** BEST1. **(A)** Upper panel: representative depiction of irregular read coverage in each sample over the entire FN1 transcript. Lower panel: The only common peak of read coverage over all samples, spanning less than 100 bp, was identified in a region of the FN1 gene annotated as intronic. **(B)** Three peaks shared by 11 out of 12 samples were identified in the BEST1 transcript read coverage, two of which were located in a gene region annotated as intronic. Selected genomic regions according to GRCh38 are shown per gene, with RefSeq annotated gene structure at the bottom of each panel. Aligned reads were mapped to the annotated gene for each EV sample and coverage is shown in grey.


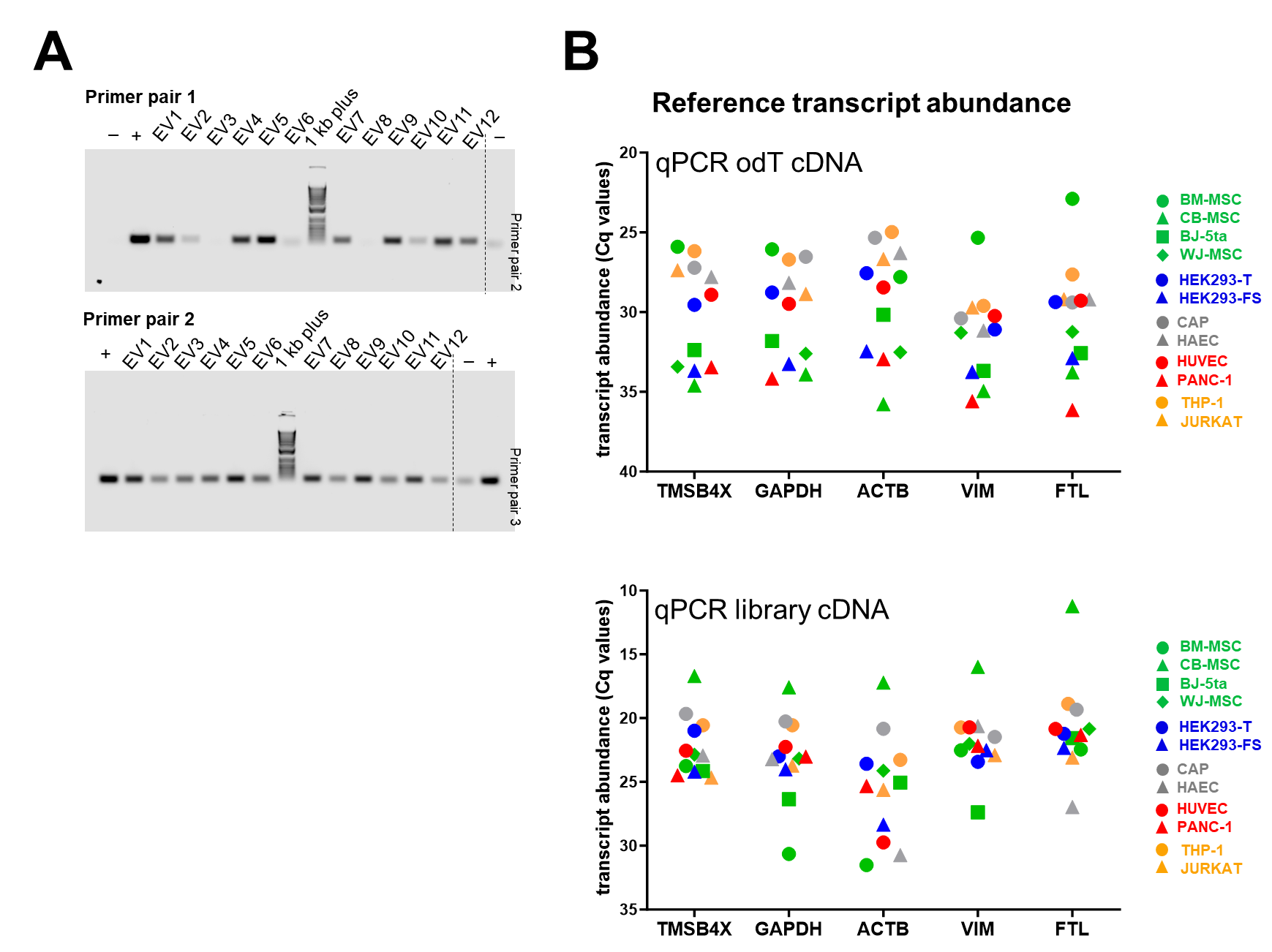


**Supplementary Figure S 4**: Validation of candidate transcript expression by RT-qPCR. **(A)** Representative agarose gel picture with PCR-products amplifed by two different primer pairs (here Primer pair 1 and 2) from cDNA transcribed from EV RNA isolated from the 12 differnent EV types (EV1 – EV 12) with negative (no template) and positive (HEK293T cell RNA-transcribed cDNA) control for amplification. Primer 1 exemplifies a primer pair not chosen for further evaluation by qPCR, while primer pair 2 was selected for further application in qPCR. **(B)** Reference transcript abundance depicted as Cq values per gene and EV type. Oligo dT-primed cDNA for qPCR was either freshly prepared (top panel, odT – oligo dT) or previously prepared as library for RNA-seq (bottom panel).


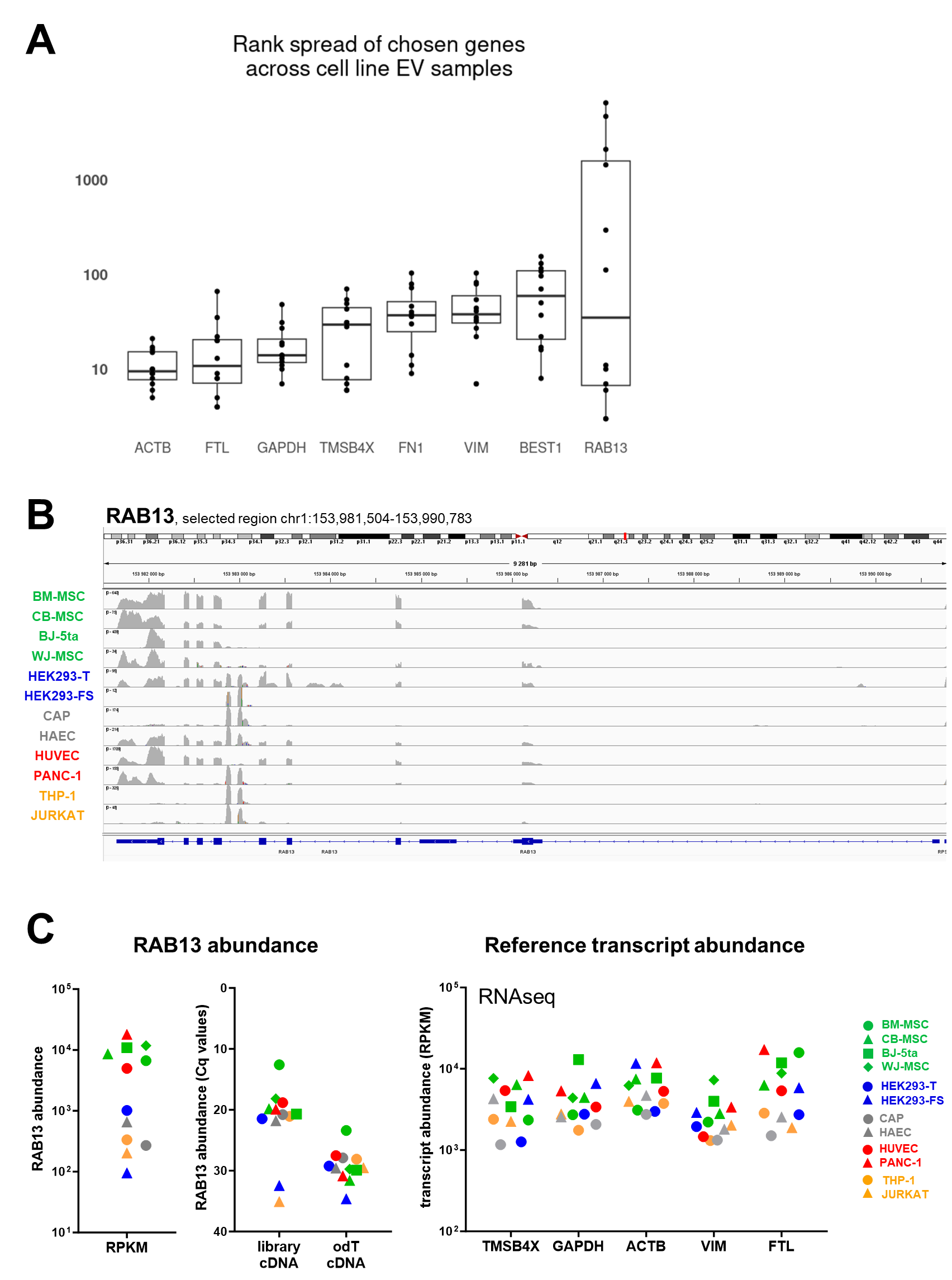


**Supplementary Figure S 5**: RAB13 as a transcript of interest for proof-of-concept normalization by five candidate reference transcripts. **(A)** Comparative rank spread across all EV types for the seven initially identified candidate reference genes (ACTB, FTL, GAPDH, TMSB4X, FN1, VIM, BEST1) and RAB13. **(B)** RAB13 read coverage of all 12 EV samples. Selected genomic regions according to GRCh38 are shown at the top, and RefSeq annotated gene structure at the bottom of the panel. Aligned reads were mapped to RAB13 for each EV sample and coverage is shown in grey, visualizing full coverage at different expression levels depending on the EV type. **(C)** Transcript abundance visualization: RAB13 abundance as measured by RPKM values (left panel) or qPCR (Cq values, middle panel), and reference transcript abundance measured by RPKM values per candidate reference gene per EV sample (right panel). odT – oligo dT-primed.


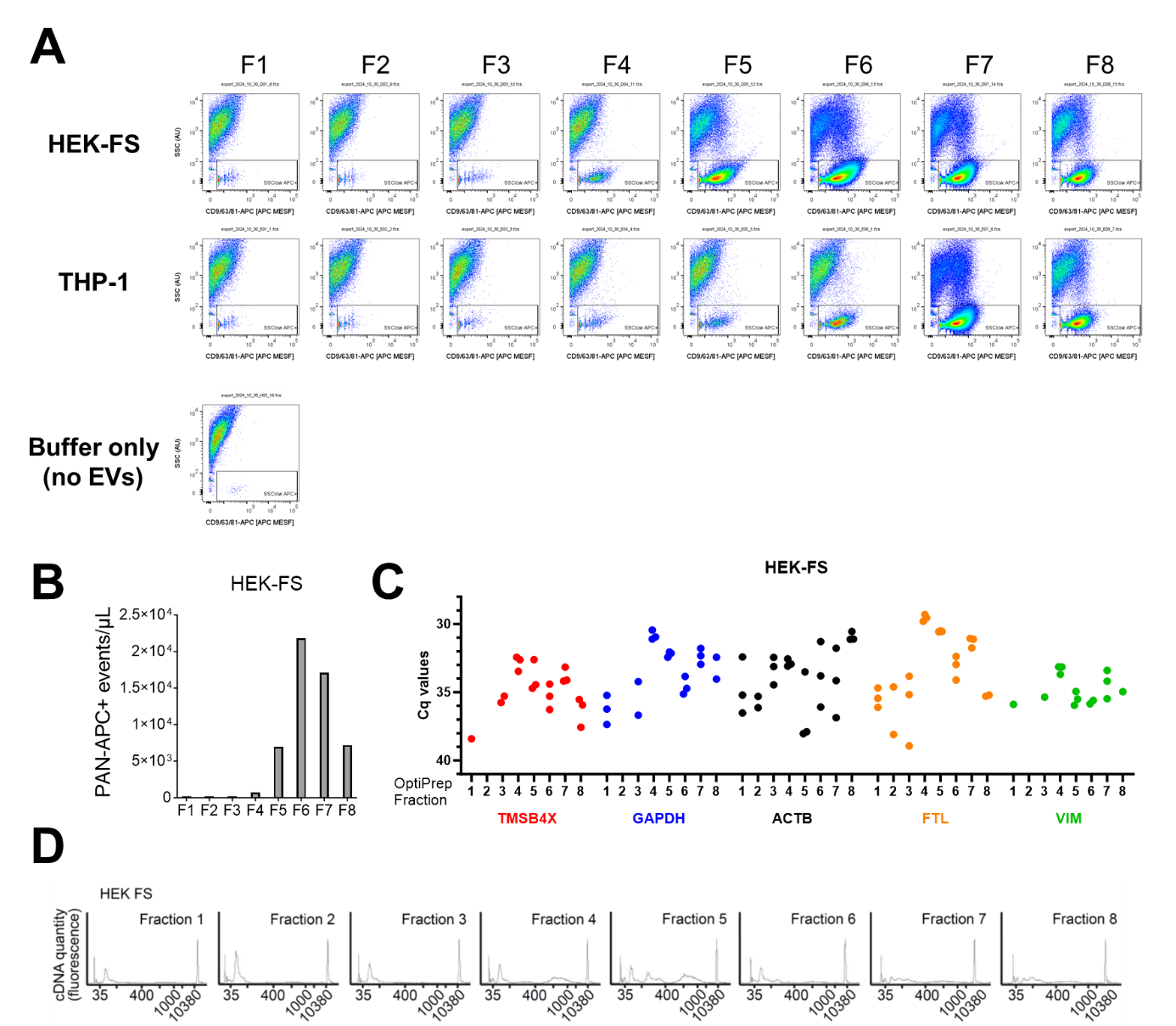


**Supplementary Figure S 6:** EV mRNA transcripts are highly abundant in OptiPrep density gradient-purified EVs. **(A)** Detailed flow cytometry plots showing the strategy for EV flow cytometry assay analysis detecting the characteristic tetraspanin protein signature (“PAN-APC” antibody mix: CD9, CD63, and CD81-antibodies labelled with APC) in all 8 fractions harvested by OptiPrep density gradient centrifugation from THP-1 and HEK293-FS EVs. **(B)** Concentration of events per µl from fractions isolated from HEK293-FS EVs as measured by EV flow cytometry. The majority of all events was detected in fraction 6 and 7. **(C)** RT-qPCR analysis of the five candidate reference transcripts, TMSB4X, GAPDH, ACTB, FTL, and VIM, in fractions 1-8 harvested from HEK293-FS EVs. **(D)** Full-length Smart-seq3 cDNA profiles run on a Bioanalyzer 2100 High Sensitivity chip.

**
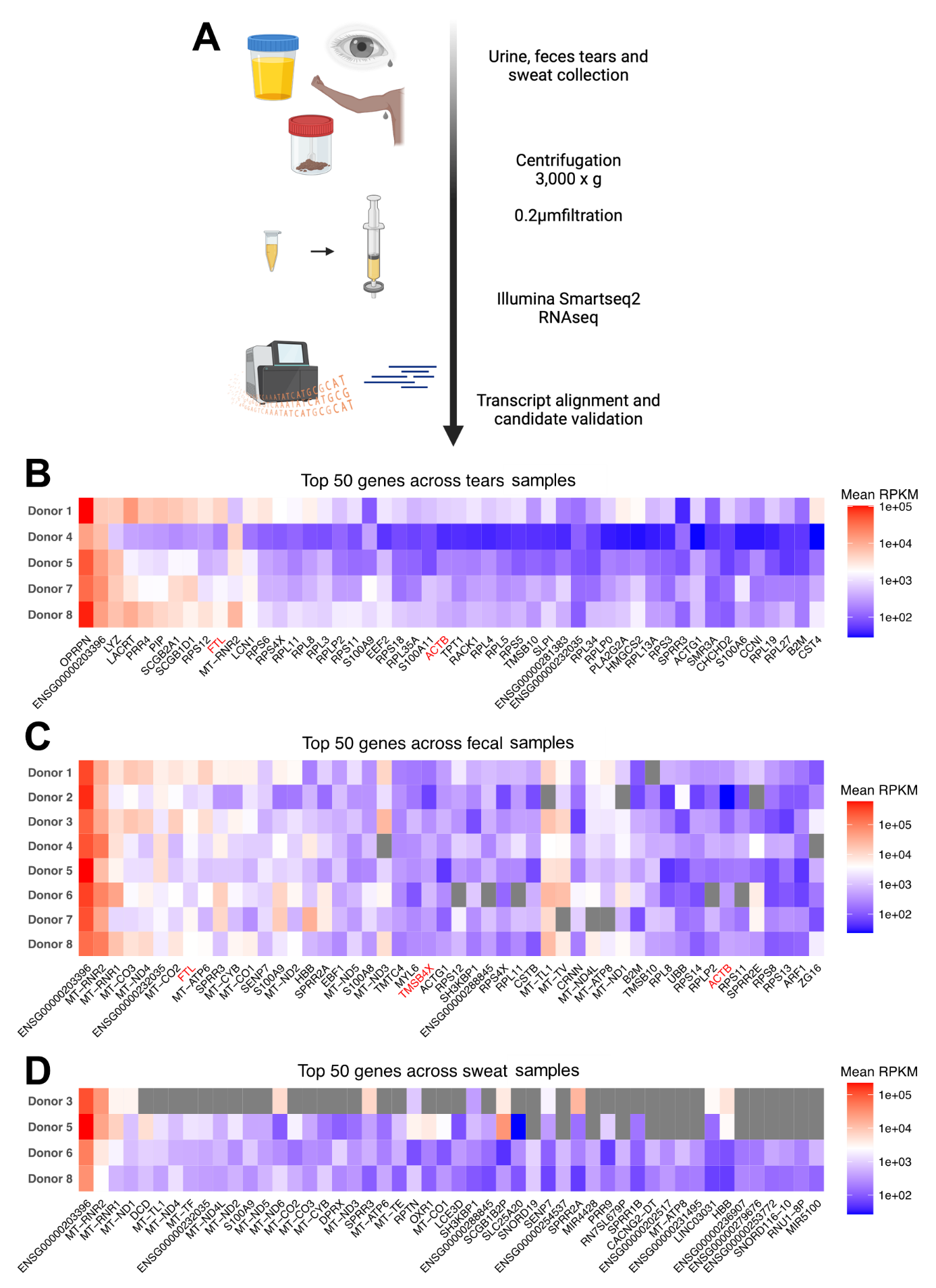
**

**Supplementary Figure S 7:** Reference transcripts are broadly expressed in minimally processed biofluids. **(A)** Workflow schematic depicting the isolation of biofluids from healthy donors, followed by RNA-seq analysis. Figure created using BioRender. **(B-D)** Two of the reference transcripts, FTL and ACTB, as well as TMS4BX in feces, (all marked in red font), are detected among the rank-sum ordered top 50 transcripts in minimally processed tears and feces from four to eight healthy donors. None of the reference transcripts are among the most abundant transcripts in sweat.
